# Exploring the Anti-Inflammatory Activity of the Heat-Processed *Gynostemma pentaphyllum* Extract (Actiponin^®^) in RAW264.7 Cells and Carrageenan-Induced Rat Models

**DOI:** 10.3390/ijms26189145

**Published:** 2025-09-19

**Authors:** Seul Ah Lee, Bo Ra Moon, Chan Hwi Lee, Sun Hee Lee, Eunju Do, Do Kyung Kim, Tae-Lin Huh, Chun Sung Kim

**Affiliations:** 1 Department of Oral Biochemistry, College of Dentistry, Chosun University, Gwangju 61452, Republic of Korea; 2 TG Biotech Research Institute, Technobuilding, Kyungpook National University, 47, Gyeongdae-ro 17-gil, Buk-gu, Daegu 41566, Republic of Koreaejdo2302@tgbio.com (E.D.); 3 Department of Oral Biology, College of Dentistry, Chosun University, Gwangju 61452, Republic of Korea; kdk@chosun.ac.kr

**Keywords:** anti-inflammatory, carrageenan-induced paw edema, *Gynostemma* *pentaphyllum*, MAPKs, NF-κB

## Abstract

*Gynostemma pentaphyllum* (GP) is a medicinal plant that has long been used as drug for the treatment of rheumatism, liver disease, and diabetes. In this study, GP was extracted with 50% ethanol extract, and then the extract was heat-processed under high pressure to analyze the anti-inflammatory potential of these extract (named actiponin (AP)) and its derived components, damulin A and damulin B, in RAW264.7 cells and carrageenan-induced rat models. Ap had no effect on RAW264.7 cells up to 180 μg/mL, but DA and DB showed cytotoxicity from 18 μM. Pretreatment with AP significantly suppressed the LPS-induced increase in nitric oxide (NO) and inducible nitric oxide synthase (iNOS) protein expression via griess reagent and Western blot analysis, and these effects were similar to those of DA and DB. AP, DA, and DB also significantly suppressed the expression of prostaglandin E2 (PGE2) and cyclooxygenase-2 (COX-2) protein, which were increased by LPS, in a concentration-dependent manner. In addition, AP, DA, and DB inhibited the LPS-induced increase in pro-inflammatory cytokines, tumor necrosis factor (TNF)-α, and interleukin (IL)-6 in RAW264.7 cells. The anti-inflammatory activities of AP, DA, and DB are mediated by the suppression of the nuclear factor (NF)-κB and phosphorylation of mitogen-activated protein kinases (MAPKs) signaling pathways. Oral administration of 30, 50, 100, or 200 mg/kg (AP) suppressed carrageenan-induced edema in a concentration-dependent manner. Collectively, these results suggest that AP exerts potential anti-inflammatory activity by suppressing the inflammatory-mediators and pro-inflammatory cytokines via the NF-κB and MAPK pathways in vitro and by reducing the thickness of carrageenan-induced paw edema in vivo.

## 1. Introduction

Consuming medicinal plants to prevent and treat illness and maintain good health is an age-old practice [1]. *Gynostemma pentaphyllum* (Thunb.) Makino (GP), a perennial herb belonging to the Cucurbitaceae family, has been used as a medicinal herb to treat various diseases, including rheumatism and diabetes, since the Ming dynasty [2]. GP has long been used in various Asian countries, including Korea, China, Japan, and Vietnam, as a traditional tea or herbal medicine [2,3]. It contains more than 20 active ingredients, including dammarane saponins, polyphenols, flavonoids, and phytosterols, and has been reported to have pharmacological activities, such as antihyperlipidemic, anticancer, hepatoprotective, antiatherogenic, neuroprotective and hypoglycemic effects [2,3,4,5,6,7,8,9,10]. The anti-inflammatory effect of gypenoside, known as the main active ingredient of GP, has been reported to improve anxiety and depression in anxious and depressed mice, and to significantly suppress the growth and glycolysis of gastric cancer by targeting the Hippo pathway [2,8,10,11]. In addition, polysaccharides derived from GP (GPP) have also been recently reported to have various pharmaceutical properties. The molecular structure of GPP contains abundant monosaccharides, such as rhamnose, xylose, and arabinose, as well as 35–40% acidic polysaccharide components, and this structure has been reported to exhibit excellent efficacy in immunomodulation, antioxidant and anti-inflammatory effects, gut microbiota regulation, and liver protection while maintaining very low pharmacological toxicity [6]. The heat-processed GP extract, named “Actiponin^®^”, was approved as a functional ingredient for health functional foods by the Ministry of Food and Drug Safety of Korea in 2013. Actiponin^®^ (AP) contains ten times the amount of damulin A (DA) and damulin B (DB), active ingredients with anti-obesity and anti-arthritics effects, compared to the standard extracts of GP [12,13]. Our previous studies reported that DA and DB are the active ingredients of AP that improve obesity by inducing AMP-activated protein kinases [12]. In addition, it was recently reported that AP and AP-derived active ingredients DA and DB can exhibit chondroprotective properties by inhibiting the activity of cartilage matrix-degrading enzymes [13]. The physiological activity of DA has not yet been reported; however, DB has been reported to promote hair growth and protect against cisplatin-induced nephrotoxicity [14,15]. Therefore, we analyzed the anti-inflammatory efficacy and mechanism of action of AP and its functional components, DA and DB.

Inflammation is an immune response that occurs when the body fights against endogenous and exogenous stimuli to protect itself [16]. However, because inflammatory responses that persist even after antigen removal can lead to autoimmune diseases, such as inflammatory arthritis and asthma, regulating inflammation at this stage is crucial for preventing disease and maintaining homeostasis in the human body [16,17]. Inflammation is mediated by elevated expression of inducible nitric oxide synthase (iNOS), nitric oxide (NO), cyclooxygenase (COX)-2, and pro-inflammatory cytokines secreted by inflammatory cells [16,18]. Macrophages are plastic cells that can switch between M1 (related immune, pro-inflammatory) and M2 (related anti-inflammatory) phenotypes depending on specific environmental conditions. In infected tissues, macrophages are initially polarized to M1, which helps clear antigens via an inflammatory response, but subsequently polarize to M2, which provides an anti-inflammatory response to heal the damaged tissue [19,20]. Lipopolysaccharide (LPS) is a potent endotoxin that polarizes macrophages toward the M1 phenotype, mimicking the early stages of the inflammatory response [18,19]. M1-stage macrophages activate transforming growth factor-β-activated kinase-1 (TAK1) via MYD88-dependent TLR4, and then TAK1 activates the mitogen-activated protein kinases (MAPKs) and IKK complexes, thereby inducing the expression of inflammatory factors [18,21,22]. Therefore, the suppression of these signaling pathways in LPS-activated macrophages plays a key role in anti-inflammatory responses and has been widely studied as a mechanism for anti-inflammatory effects [21,23,24].

To investigate the anti-inflammatory effects and mechanisms of AP, DA, and DB, we used LPS-induced RAW264.7 and carrageenan-induced paw edema in rats. In RAW264.7 cells, AP, DA, and DB significantly suppressed the expression of iNOS, COX-2, TNF-α, and IL-6, which were increased by LPS, and we confirmed that these effects may involve MAPK and NF-κB signaling pathways. In addition, in a carrageenan-induced paw edema inflammation model, AP was administered for 4 h and showed a significant inhibition of edema over time in a concentration-dependent manner.

## 2. Results

### 2.1. Effect of AP, DA, and DB on Viability of RAW264.7 Cells

**Figure 1:**
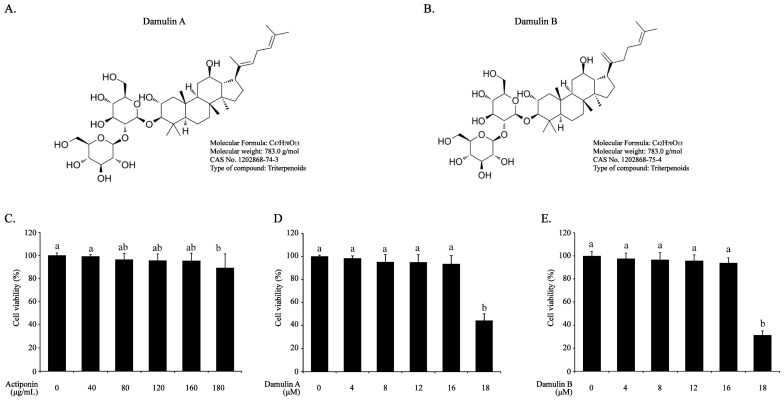
Effects of AP, DA, and DB on viability of RAW264.7 cells. (**A**) Chemical formular of DA. (**B**) Chemical formular of DB. The cells (2.5 × 10^5^ cells/well) were cultured in 48-well plates and treated with AP, DA, and DB at the indicated concentrations for 24 h. Thereafter, cell viability of (**C**) AP, (**D**) DA, and (**E**) DB was analyzed using the CCK-8 as-say and graphed as a percentage. Data are presented as the mean ± SEM of three independent experiments. a–b Mean values designated by different superscript letters were significantly different between groups at *p* < 0.05, as determined by Duncan’s multiple-range test.

The cytotoxicity of AP, DA, and DB on RAW264.7 cells was analyzed using the CCK-8 assay. AP had no effect on cell viability up to 200 μg/mL, whereas DA and DB had no effects up to 16 μM (Figure 1). Therefore, to avoid confounding efficacy owing to cytotoxicity, all subsequent experiments were performed at an AP concentration below 160 μg/mL and DA and DB concentrations below 16 μM.

### 2.2. Inhibitory Effect of AP, DA, and DB on the LPS-Induced NO Production and iNOS Expression in RAW264.7 Cells

**Figure 2:**
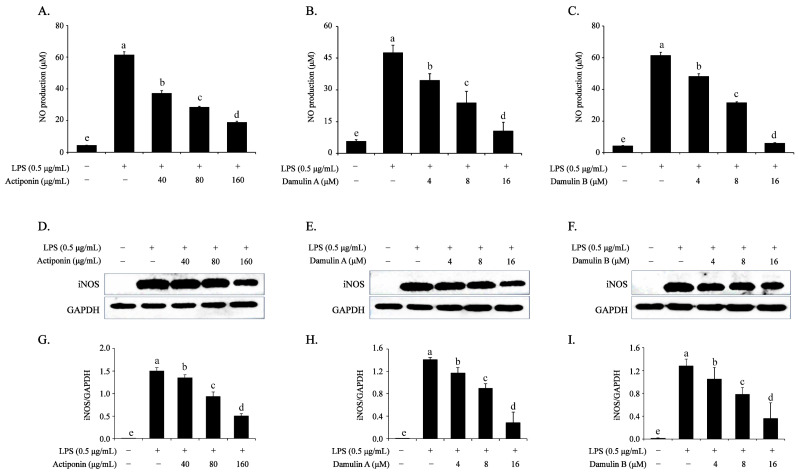
Effects of AP, DA, and DB on NO production and iNOS expression in LPS-stimulated RAW264.7 cells. The cells (2.5 × 10^5^ cells/well in 48-well plate for NO assay, 2 × 10^6^ cells/100 mm dishes for Western blot) are cultured and treated with AP, DA, and DB for 4 h, then incubated with LPS (500 ng/mL) for another 20 h. Nitrite accumulated in the culture medium is analyzed using Griess reagent, and calibration curve is based on NaNO_3_; (**A**) AP, (**B**) DA, and (**C**) DB. Protein expression of iNOS is determined using Western blot analysis; (**D**) AP, (**E**) DA, (**F**) DB. (**G**–**I**) Quantitative data of (**D**–**F**) are analyzed using ImageJ bundle with Java 1.8.0_172 software. GAPDH served as an internal control. The data are presented as the mean ± SEM of three independent experiments. a–e Mean values designated by different superscript letters are significantly different between groups at *p* < 0.05 as determined by Duncan’s multiple-range test.

To analyze the anti-inflammatory effects of AP, DA, and DB, LPS was used to stimulate the release of NO and iNOS from macrophages to indirectly create a chronic inflammatory environment. The LPS group showed significantly higher NO production than the control group (Figure 2A–C). However, pretreatment with AP effectively prevented LPS-induced NO production (61.40 ± 0.48 μM), with inhibition rates of 39% (37.24 ± 0.28 μM), 54% (28.42 ± 0.19 μM), and 69% (18.89 ± 0.14 μM) at 40, 80, and 160 μg/mL, respectively (Figure 2A). DA and DB, the active ingredients of AP, showed a similar efficacy to that of AP, and pretreatment with DA and DB significantly inhibited LPS-induced NO production in a concentration-dependent manner (Figure 2B,C). To understand the mechanism underlying the inhibition of LPS-induced NO production, changes in iNOS protein expression were analyzed. Pretreatment with AP, DA, and DB effectively inhibited LPS-induced iNOS expression (Figure 2E–I). These results suggested that the inhibition of NO production was associated with the regulation of iNOS expression mediated by AP, DA, and DB in LPS-stimulated RAW264.7 cells.

### 2.3. Inhibitory Effect of AP, DA, and DB on LPS-Induced PGE2 Production and COX-2 Expression in RAW264.7 Cells

**Figure 3:**
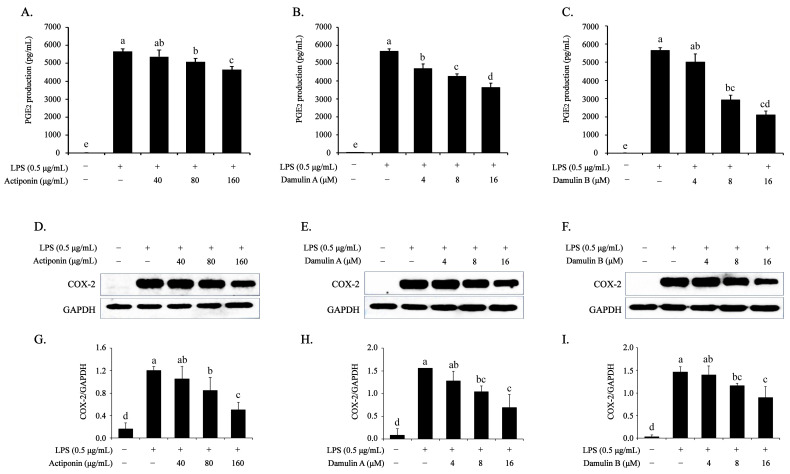
Effects of AP, DA, and DB on PGE2 production and COX-2 expression in LPS-stimulated RAW264.7 cells. The cells (2 × 10^6^ cells) are cultured in 100 mm dishes and treated with AP, DA, and DB for 4 h, then incubated with LPS (500 ng/mL) for another 20 h. PGE2 accumulated in the culture medium is analyzed using PGE2 ELISA; (**A**) AP, (**B**) DA, (**C**) DB. Protein expression of COX-2 is determined using Western blot analysis; (**D**) AP, (**E**) DA, (**F**) DB. (**G**–**I**) Quantitative data of (**D**–**F**) were analyzed using ImageJ software. GAPDH served an internal control. The data are presented as the mean ± SEM of three independent experiments. a–e Mean values designated by different superscript letters are significantly different between groups at *p* < 0.05, as determined by Duncan’s multiple-range test.

As inflammation causes itching and pain, changes in PGE2 production and COX-2 expression induced by AP, DA, and DB were analyzed. LPS alone significantly increased the PGE2 production (5656.64 ± 51.23 pg/mL); however, pretreatment with AP prevented this effect, showing a 18% inhibition rate at 160 μg/mL (4640.96 ± 53.18 pg/mL) (Figure 3A). Similarly to the inhibitory effect of AP on PGE2 production, pretreatment with DA and DB effectively inhibited LPS-induced PGE2 production, with inhibition rates of 36% (3635.20 ± 55.18 pg/mL) and 62% (2101.43 ± 48.14 pg/mL), respectively, at 16 μM (Figure 3B,C). To understand the mechanism by which these compounds inhibit LPS-induced PGE2 production, changes in the protein expression of COX-2, which synthesizes PGE2, were analyzed. Pretreatment with AP, DA, or DB significantly suppressed LPS-induced COX-2 expression (Figure 3D–I). These results suggested that the inhibition of PGE2 production was associated with the regulation of COX-2 expression mediated by AP, DA, and DB in LPS-stimulated RAW264.7 cells.

### 2.4. Inhibitory Effect of AP, DA, and DB on LPS-Induced Pro-Inflammatory Cytokines in RAW264.7 Cells

**Figure 4:**
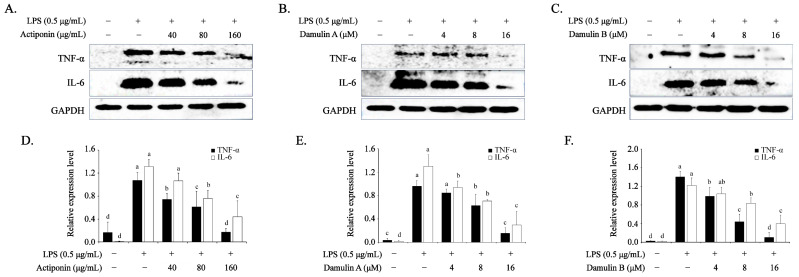
Effects of AP, DA, and DB on pro-inflammatory cytokines in LPS-stimulated RAW264.7 cells. The cells (2 × 10^6^ cells) are cultured in 100 mm dishes and treated with AP, DA, and DB for 4 h, then incubated with LPS (500 ng/mL) for another 20 h. Protein expression of TNF-α and IL-6 is determined using Western blot analysis; (**A**) AP, (**B**) DA, (**C**) DB. (**D**–**F**) Quantitative data of (**A**–**C**) are analyzed using ImageJ software. GAPDH served as an internal control. The data are presented as the mean ± SEM of three independent experiments. a–d Mean values designated by different superscript letters are significantly different between groups at *p* < 0.05, as determined by Duncan’s multiple-range test.

**Figure 5:**
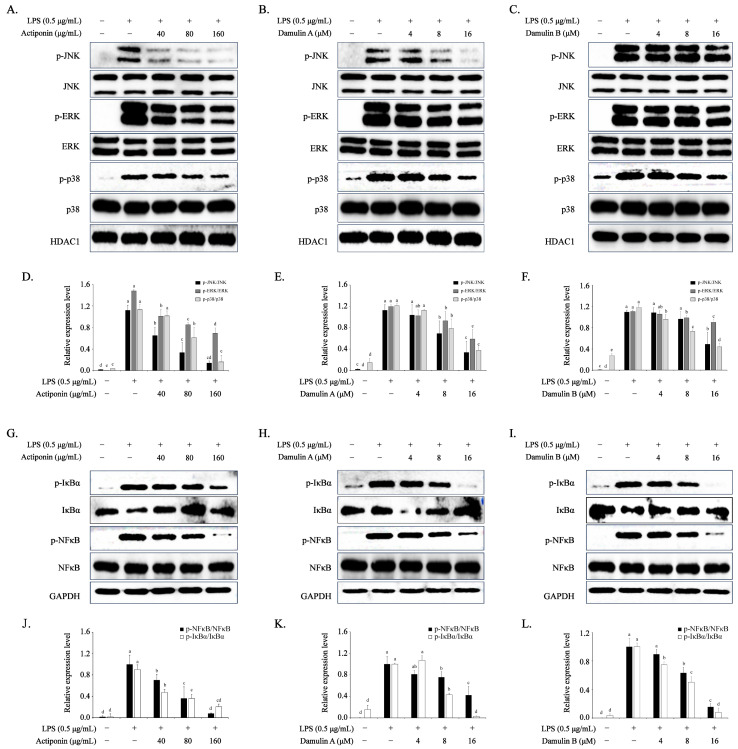
Effects of AP, DA, and DB on MAPKs and NF-κB pathways in LPS-stimulated RAW264.7 cells. The cells (2 × 10^6^ cells) are cultured in 100 mmm dishes and treated with AN, DA, and DB for 1 h, then incubated with LPS (500 ng/mL) for another 30 min. Protein expression of MAPKs (t/p-ERK, t/p-JNK, and t/p-p38) and NF-κB (t/p-IκB-α and t/p-p65) is determined using Western blot analysis. MAPKs: (**A**) AP, (**B**) DA, (**C**) DB, NF-κB (t/p-IκB-α and t/p-p65); (**G**) AP, (**H**) DA, (**I**) DB. (**D**–**F**,**J**–**L**) Quantitative data of (**A**–**C**,**G**–**I**) are analyzed using ImageJ software. GAPDH served as an internal control. The data are presented as the mean ± SEM of three independent experiments. a–e Mean values designated by different superscript letters are significantly different between groups at *p* < 0.05, as determined by Duncan’s multiple-range test.

Cytokines are important proteins that transmit intracellular signals and regulate inflammatory responses [16,17,25]. Analysis of the effects of AP, DA, and DB on pro-inflammatory cytokines showed that LPS significantly increased the expression of TNF-α and IL-6 compared to that in the control group (Figure 4); however, pretreatment with AP, DA, and DB reversed these effects in a dose-dependent manner. AP reduced TNF-α expression by 31–85% and IL-6 expression by 20–67% compared with the LPS group (Figure 5A,D). Furthermore, DA, the active ingredient in AP, suppressed TNF-α expression by 85% and IL-6 expression by 78% at 16 μM, whereas another active ingredient, DB, reduced TNF-α expression by 93% and IL-6 expression by 68% at the same concentration (Figure 5B,C,E,F). These results suggest that AP, DA, and DB significantly suppressed LPS-induced expression levels of TNF-α and IL-6 proteins, supporting the hypothesis that AP, DA, and DB have anti-inflammatory effects.

### 2.5. Inhibition of AP, DA, and DB on NF-κB Signaling Pathway in LPS-Stimulated RAW 264.7 Cells

NF-κB is a transcription factor closely related to inflammation and immune response. When NF-κB is activated, IκB-α is phosphorylated and degraded, and accordingly, the NF-κB subunit p65 is phosphorylated and translocated to the nucleus, where it regulates the expression of inflammation-related factors such as iNOS, COX-2, NO, PGE2, TNF-α, and IL-6. Therefore, to evaluate the molecular mechanism underlying the inhibitory effects of AP, DA, and DB on inflammatory-related factors, we analyzed the phosphorylation of IκB-α and p65. LPS stimulation for 30 min increased the phosphorylation of IκB-α and boosted the phosphorylation of p65 (Figure 5A–F). However, pretreatment with AP, DA, and DB for 1 h prevented IκB-α from being phosphorylated and degraded and suppressed the phosphorylation of p65 in a dose-dependent manner (Figure 5A–F). The results suggest that AP, DA, and DB inhibit the activation of NF-κB by preventing the phosphorylation and degradation of IκB-α in LPS-stimulated RAW264.7 cells (Figure 5).

### 2.6. Inhibition of AP, DA, and DB on MAPKs in LPS-Stimulated RAW 264.7 Cells

In eukaryotes, MAPKs consist of ERK, JNK, and p38, which regulate the expression of inflammation-related cells. To determine whether the inhibitory effects of AP, DA, and DB on LPS-induced inflammation-related factors were mediated through the MAPK signaling pathway, we analyzed the effects of AP, DA, and DB on the LPS-induced phosphorylation of ERK, JNK, and p38 in RAW 264.7 cells by Western blot. LPS stimulation for 30 min increased the phosphorylation of ERK, JNK, and p38 (Figure 5G–L). However, pretreatment with AP, DA, or DB for 1 h dose-dependently inhibited the phosphorylation of ERK, JNK, and p38 without affecting the total amount of each MAPK (Figure 5G–L). These results suggest that the inhibitory actions of AP, DA, and DB on MAPK activation may be the underlying mechanism of their anti-inflammatory effects.

### 2.7. Effects of AP on Carrageenan-Induced Rat Paw Edema

**Figure 6:**
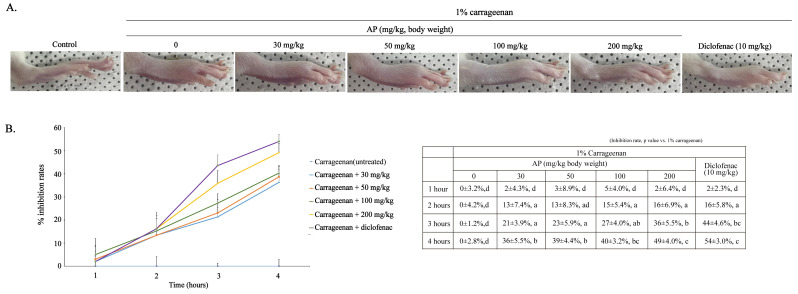
Effects of AP on carrageenan-induced paw edema. Paw edema is induced by subcutaneous injection of a 1% carrageenan solution (100 uL/animal) into the plantar area of rats, followed by oral administration of AP and diclofenac (10 mg/kg) 1 h later. (**A**) Morphological characteristics of the paw of rats 4 h after carrageenan injection. (**B**) Paw thickness is measured using digital calipers each hour after carrageenan injection for a total of 4 h. The data are presented as the mean ± SEM of four animals. a–d Mean values designated by different super-script letters are significantly different between groups at *p* < 0.05, as determined by Duncan’s multiple-range test.

The anti-inflammatory effects of AP were evaluated 4 h after carrageenan injection in a mouse model of carrageenan-induced paw edema. Compared to the control group, the group injected with carrageenan alone showed a significant increase in paw thickness, whereas the experimental group, to which AP (30, 50, 100, and 200 mg/kg body weight) was orally administered 1 h prior, showed a concentration- and time-dependent decrease in paw thickness (Figure 6). The slope of the edema inhibition rate by AP rapidly in-creased 2 h after carrageenan injection. In the 30, 50, 100, and 200 mg/kg groups, the inhibition rates of edema were 13%, 13%, 15%, and 16%, respectively, at 2 h, and 36%, 39%, 40%, and 49%, respectively, at 4 h (Figure 6B). The edema-inhibitory effect in the 200 mg/kg group was similar to that in the positive control group at 4 h (Figure 6B). The morphological characteristics 4 h after carrageenan injection are illustrated in Figure 6A. These results suggest that oral administration of AP at concentrations of 30, 50, 100, and 200 mg/kg significantly inhibited carrageenan-induced paw edema. These results suggest that the oral administration of AP has an anti-inflammatory effect, as verified by its inhibitory effect on carrageenan-induced paw edema.

## 3. Discussion

Inflammation is the most basic physiological response of the human body and is triggered by the phagocytosis of immune cells to protect the body from external or internal irritants. Inflammatory responses that cannot be adequately controlled after the removal of irritants are potential risk factors for various diseases and are known to accelerate the onset of cancer and colon, brain, and joint diseases [16]. Therefore, suppression of inflammation offers a strategy for preventing secondary diseases caused by excessive inflammatory responses. Nonsteroidal anti-inflammatory drugs (NSAIDs) are primarily used to relieve inflammatory symptoms [16]. These drugs have excellent efficacy but also have unavoidable side effects [26]. Therefore, medicinal plants that do not have significant side effects, even when consumed for a relatively extended period, are expected to serve as alternatives to existing anti-inflammatory drugs.

Gynostemma pentaphyllum (GP), a medicinal plant used to treat rheumatism and diabetes [2]. In our previous study, we demonstrated that heat-treated GP extract (named Actiponin^®^, AP) alleviates obesity through AMP-activated protein kinase (AMPK) activity, and that this effect is mediated by damulin A and damulin B [12,27]. The anti-obesity efficacy of AP in in vitro and in vivo models was examined in a randomized, double-blind, placebo-controlled trial in obese subjects, in which supplementation for 12 weeks significantly reduced the total abdominal fat area and body weight without any significant side effects [27]. Despite these benefits, there is a lack of research on its anti-inflammatory effects and underlying mechanisms. This study evaluated the anti-inflammatory efficacy of AP in LPS-stimulated RAW264.7 and carrageenan-induced paw edema.

Macrophages are distributed throughout all body tissues and are involved in the initiation or delay of several diseases, including infections, immunity, inflammation, and cancer [19,28]. Macrophages activated by stimuli such as LPS promote the secretion of inflammatory mediators and pro-inflammatory cytokines, such as iNOS, PGE2, NO, COX-2, TNF-α, and IL-6 [16,19,29]. Overexpression of these inflammation-related factors leads to further inflammation, persistently maintaining the inflammatory environment [17,25,26]. Among these, TNF-α and IL-6 are cytokines and chemokines that recruit neutrophils and macrophages induce the secretion of other cytokines, thereby initiating and enhancing inflammation [17,26]. Therefore, targeting their expression promises significant anti-inflammatory effects; for this reason, their expression is considered a parameter for evaluating anti-inflammatory effects [14,18,21]. In this study, AP, DA, and DB significantly inhibited the expression of NO, PGE2, iNOS, COX-2, TNF-α, and IL-6 in LPS-stimulated RAW264.7 cells. AP, DA, and DB significantly suppressed TNF-α and IL-6 in a concentration-dependent manner in LPS-stimulated RAW264.7 cells, indicating their definite efficacy in suppressing inflammatory responses. MAPK and NF-κB are representative signal transduction systems involved in the overall physiological activity of cells [22,30,31]. Activation through phosphorylation of MAPKs (ERK, JNK, and p38) is essential for the release of inflammatory cytokines and chemokines [21,22]. Additionally, under normal circumstances, the activity of the NF-κB subunit p65 is suppressed by IκB-α in the cytoplasm; however, under inflammatory circumstances, IκB-α is phosphorylated and p65 released from the complex translocates to the nucleus, where it induces the expression of inflammation-related factors, such as NO, iNOS, PGE2, COX-2, TNF-α, and IL-6 [30,31,32]. Our results showed that AP, DA, and DB prevented the nuclear translocation of p65 by inhibiting the phosphorylation of IκB-α, and also inhibited the phosphorylation of MAPKs (ERK, JNK, and p38), without affecting the total expression level. These results indicate that the inhibitory effect of AP on the expression of inflammation-associated factors may be partially mediated through NF-κB and MAPK signaling. These results are consistent with the anti-inflammatory efficacy mechanism of natural resources. Zhu reported that the inhibitory effect of ophioglossum vulgatum-derived ophioglonin on the expression of iNOS, COX-2, TNF-α, IL-1β, and IL-6 is mediated through the inhibition of the NF-κB and MAPK signaling pathways [21,33].

To verify the anti-inflammatory effect of AP, edema was induced in the paws of rats using carrageenan, and the alleviation of paw edema was analyzed. Carrageenan-induced paw edema is typically used as an in vivo model to evaluate anti-inflammatory effects [34]. Carrageenan-induced inflammation occurs as a two-phase inflammatory response that lasts up to 4 h, with histamine, bradykinin, and cyclooxygenase produced 2 h after carrageenan administration, followed by the production of arachidonic acid metabolites between 2 and 4 h [34,35,36,37,38,39]. Anti-inflammatory studies using carrageenan-induced paw edema generally analyze the results up to 6 h after carrageenan injection. However, because the carrageenan-induced inflammatory response cannot clearly explain the inflammatory response after the first 4 h despite the effects of kinins, complement, etc., this study conducted a follow-up study up to 4 h, as in previous studies [37,38,39,40,41]. In the in vivo study, oral administration of AP gradually inhibited carrageenan-induced paw edema in a time- and concentration-dependent manner, and the slope of the inhibition rate increased from 2 h onward. These results indicate that AP inhibited arachidonic acid-induced inflammatory responses more effectively than histamine-induced inflammatory responses.

Despite finding beneficial anti-inflammatory effects of AP, DA, and DB, this study has several limitations. First, the efficacy of AP, DA, and DB was confirmed through pretreatment. The pretreatment method was chosen because this study was focused on the preventive level, like health-functional foods. However, to achieve a broader therapeutic effect, it is necessary to establish scientific evidence for the anti-inflammatory effects of simultaneous and post-treatment in the future. It is also believed that further research is needed to clarify the mechanisms underlying the anti-inflammatory effects of DA and DB. DA and DB are pure compounds that have potential for development into future pharmaceuticals. In this study, we confirmed through protein expression analysis that they contain MAPK and NF-κB signaling. However, if we can provide a clear mechanism through research into which site they directly or indirectly induce activity, they will be highly valuable as new drug candidates.

## 4. Materials and Methods

### 4.1. Reagents

Sulfanilamide (CAS No. 63-74-1), N-(1-naphthyl) ethylenediamine dihydrochloride, phosphoric acid (CAS No. 1465-25-4), carrageenan (CAS No. 9064-57-7), lipopolysaccharide (LPS, CAS No. 93572-42-0), and 3-(4,5-dimethylthiazol-2-yl)-2,5-diphenyltetrazolium bromide (MTT, CAS No. 298-93-1) were purchased from the Sigma-Aldrich Corporation (St. Louis, MO, USA). Prostaglandin E2 (PGE2) enzyme-linked immunosorbent assay (ELISA) kit was purchased from R&D Systems (Minneapolis, MN, USA). Antibodies against iNOS (#13120), COX-2 (#4842S), TNF-α (#3707), ERK (#9102), p-ERK (#9101), JNK (#9252), p-JNK (#9251), p38 (#9212), p-p38(#9211), GAPDH (#2118), and HDAC1 (#5356) were purchased from Cell Signaling Technology (CST, Danvers, MA, USA). Antibodies against NF-κB (PA5-16545), p-NF-κB (MA5-15160), IκB-α (MA5-15132), and p-IκB-α (MA5-15224) were purchased from Invitrogen (Thermo Fisher Scientific Inc., Waltham, MA, USA). The IL-6 antibody was purchased from Abcam (Cambridge, UK). Secondary anti-mouse and anti-rabbit horseradish peroxidase-conjugated IgG antibodies were purchased from Genetex (Irvine, CA, USA) and CST, respectively. Dulbecco’s Modified Eagle’s medium-high glucose (DMEM-HG), penicillin-streptomycin (P/S), and fetal bovine serum (FBS) were purchased from Gibco (Waltham, MA, USA). The Cell Counting Kit-8 (CCK-8) was purchased from Dojindo (Kuma-moto, Japan). Griess reagent was purchased from BIOMAX (Gyeonggi-do, Republic of Korea).

### 4.2. Preparation of GP Extract (Actiponin ^®^), DA, and DB

AP, DA, and DB extracts were prepared according to the method described by Nguyen et al. [12,27,39]. To prepare AP, dried GP leaves were washed and subsequently undergo extraction with 8 volumes (*v*/*w*) of 50% ethanol twice. The extracts were pooled and subjected to heating under high pressure and temperature. After 2.5 to 4.0 h of heating, the extract concentrate was spray-dried with maltodextrin to form a fine powder and packaged. DA and DB were purified from the heat-treated extract of GP leaves by HP-20 ion exchange resin column chromatography (20 × 65 cm), as previously described [12], and the pure compounds were analyzed by HPLC (Shimadzu (CBM-40) System, San Jose, CA, USA) [12,13].

### 4.3. Cell Culture and Viability Assay

RAW 264.7 murine macrophage cells were purchased from the Korean Collection for Type Cultures (Daejeon, Republic of Korea). The cells were cultured in DMEM-HG containing 10% FBS and 1% P/S at 37 °C in a humidified incubator with 5% CO_2_. Cells (2.5 × 10^5^ cells/well) were seeded in 48-well plates and incubated for 24 h. After incubation, the cells were treated with AP (0, 40, 80, 120, 160, and 200 μg/mL), DA (0, 4, 8, 12, 16, and 20 μM), and DB (0, 4, 8, 12, 16, and 20 μM) in serum-free DMEM-HG for 24 h. Cell viability was assessed using the CCK-8 assay according to the manufacturer’s instructions. CCK-8 solution (10 μL of reagent in 100 μL of culture medium) was added to each well and incubated for 2 h. Absorbance was measured at 450 nm using a microplate spectrophotometer (MOBI, Seoul, Republic of Korea). The viability of the control cells was set to 100%, and the viability relative to the control was shown.

### 4.4. NO and PGE2 Production Assay

RAW 264.7 cells (2.5 × 10^5^ cells/well) were seeded in 48-well culture plates and incubated for 24 h. After incubation, the cells were treated with AP, DA, or DB in serum-free DMEM-HG. Following 4 h of treatment, the cells were stimulated with LPS (0.5 μg/mL) for 20 h. Nitric oxide (NO) concentration was measured using Griess reagent according to the manufacturer’s protocol. After incubation, 100 μL of Griess reagent was added to 100 μL of the supernatant in a 96-well plate. After 10 min of incubation, the absorbance was measured at 540 nm using a microplate spectrophotometer. The nitrite concentration was calculated using a calibration curve generated from a standard nitrite solution. PGE2 accumulation in the culture medium was quantified using a prostaglandin E2 assay kit according to the manufacturer’s protocol, and the absorbance was measured at 450 nm using a microplate spectrophotometer.

### 4.5. Protein Extraction and Western Blot Analysis

For cytokine protein expression analysis, RAW 264.7 cells (2 × 10^6^) were seeded in 100 mm dishes and incubated for 24 h. After incubation, cells were treated with AP, DA, or DB in serum-free DMEM-HG. Following 4 h of treatment, the cells were stimulated with LPS (0.5 μg/mL) for 20 h, whereas for MAPK and NF-κB activity analysis, cells were pretreated with the AP, DA, and DB for 1 h and then, co-stimulated with LPS for 30 min. Total proteins were extracted by lysing the cells with ProEXTM CETi lysis buffer (TransLab, Daejeon, Republic of Korea) for 30 min on ice, followed by centrifugation at 16,000× *g* for 15 min at 4 °C. The protein concentration was quantified using a BCA assay (Thermo Fisher Scientific Inc.) according to the manufacturer’s protocol, with a standard curve generated using BSA (Thermo Fisher Scientific Inc.). Equal amounts of protein (20 μg) were denatured at 95 °C, separated on 8–12% sodium dodecyl sulfate polyacrylamide gels, and transferred onto polyvinylidene fluoride membranes. After reacting with a 5% skimmed milk solution for 20 min to prevent non-specific binding, the membrane was incubated with specific primary antibodies overnight at 4 °C. After washing three times with Tris-buffered saline containing Tween-20, the membranes were incubated with horseradish peroxidase-conjugated secondary antibodies for 1 h. Immunoactive protein bands were detected using a chemiluminescence kit (Thermo Fisher Scientific Inc.) and visualized using Azure 300 (Azure Biosystems, Dublin, OH, USA).

### 4.6. Animals Used in the Study

Specific pathogen-free Sprague Dawley rats (male, weighing 300–320 g) were purchased from Damool Science (Daejeon, Republic of Korea) and maintained in environ-mentally controlled plastic cages (temperature: 21 ± 1 °C; humidity: 55 ± 5%; 12 h light/dark cycle). All animals had access to water and chow pellets ad libitum. All animal care, experimental procedures, and sacrifices were planned and conducted according to the Guide for the Care and Use of Laboratory Animals of the National Institutes of Health (NIH) and were approved by the Institutional Animal Care and Use Committee of Chosun University (approval number: CIACUC2024-A0016). There were no data or analyses that were not included, no experimental animals were excluded from the analysis, and all experimental animals completed the experiment without any abnormal signs.

### 4.7. Inducing Edema in Rat Paws with Carrageenan

An anti-inflammatory experimental method for suppressing paw edema was developed by Akinnawo et al. [40]. A total of 28 experiment animals were randomly assigned to 7 groups of 4 each in a blind manner, and no experimental animals were included or excluded during the experiment. One hour after oral administration of AP (30, 50, 100, and 200 mg/kg) and diclofenac sodium (10 mg/kg), 0.1 mL of 1% (*w*/*v*) carrageenan was injected subcutaneously into the plantar of the experimental animals to induce paw edema, except in the control group. Thereafter, paw thickness was monitored five times every hour for 4 h using a digital caliper, and the average value was used to calculate the percentage of edema inhibition using the following formula:

Inhibition rate (%) = ((Ct − C0)control − (Ct − C0)treated)/(Ct − C0)control × 100

where

Ct = paw thickness (mm) of the left hind limb at time t.
C0 = paw thickness (mm) of the left hind limb before carrageenan injection.
(Ct−C0)control = increase in paw size after carrageenan injection at time t
(Ct−C0)treated = increase in paw size after carrageenan injection at time t of reference or test drug-treated rats.

The rats (*n* = 28) were randomly divided as follows:

Group 1 (Control) = nothing.
Group 2 (vehicle)= 1% carrageenan + vehicle (0.9% saline).
Group 3–6 (AP30–200 mg/kg body weight) = 1% carrageenan + 30, 50, 100, and 200 mg/kg body weight of AP dissolved in 0.9% saline, respectively).
Group 7 (Diclofenac) = 1% carrageenan + 10 mg/kg body weight, diclofenac dissolved in 0.9% saline).

### 4.8. Statistical Analysis

All experimental data were obtained from at least three independent experiments, with the exception of the in vivo experiments. Data were analyzed using IBM SPSS Statistics 27 (IBM Corporation, Armonk, NY, USA) and Graphpad Prism 5.0 software (GraphPad, Inc. San Diego, CA, USA). Gene expression and paw thickness data were compared using one-way analysis of variance (ANOVA), followed by Duncan’s multiple-range test and Tukey’s test, respectively. All results are expressed as the mean ± standard error of the mean (SEM). Results with *p* < 0.05 were considered statistically significant.

## 5. Conclusions

In this study, we investigated the anti-inflammatory effect and its mechanism of AP, DA, and DB in RAW264.7 cells and carrageenan-induced paw edema model. Our results demonstrated that AP, DA, and DB have anti-inflammatory effects by suppressing inflammatory-mediators and pro-inflammatory cytokine expression through the inhibition of NF-κB and MAPK signaling in the LPS-stimulated inflammatory responses. In addition, AP gradually suppressed edema over time from 1.5 to 4 h after carrageenan injection, suggesting that it may exert anti-inflammatory effects through the inhibition of inflammatory mediators such as histamine, prostaglandin, and cytokines. These results are considered remarkable compared to other natural extracts, which show anti-inflammatory effects at a later stage (3–4 h), and are thought to have great potential for the development of various anti-inflammation-based therapeutic agents in the future.

## Data Availability

All data generated or analyzed during this study are included in this manuscript and its information files.
